# Patient satisfaction with physician assistant/associate care: an international scoping review

**DOI:** 10.1186/s12960-019-0428-7

**Published:** 2019-12-27

**Authors:** Roderick S. Hooker, Amanda J. Moloney-Johns, Mary M. McFarland

**Affiliations:** 1 Health Policy Analyst, 15917 NE Union Rd, Unit 45, Ridgefield, WA USA; 2 Department of Family and Preventive Medicine, Physician Assistant Program, The University of Utah, Salt Lake City, Utah USA; 3 Eccles Health Sciences Library, The University of Utah, Salt Lake City, Utah USA

## Abstract

**Background:**

As the role of the physician assistant/associate grows globally, one question is: what is the level of patient satisfaction with PAs? Driven by legislative enactments to improve access to care, the PA has emerged as a ready and able medical professional to address workforce shortages. The aim of this study was to review the literature on patient satisfaction of PAs.

**Objectives:**

The basis for this review was to clarify working definitions, synthesize the evidence, and establish conceptual boundaries around the topic of patient satisfaction with PAs. The intent was to identify gaps in the literature and offer suggested undertakings for more clarification on the subject.

**Methods:**

A scoping review was undertaken. Literature from 1968 to 2019 was searched and filtered for eligibility. Those that met criteria were categorized by date, method, geography, themes, and design.

**Results:**

In total, there were 987 papers or reports that were identified through bibliography database searching. Additional articles found through snowball methodology-reviewing references (*n* = 11). Only English language articles emerged for analysis. From this effort, 25 articles surfaced from the filtering process for final inclusion. Most (72%) of the articles came from the United States of America, three from the United Kingdom, and one each from Ireland, the Netherlands, and New Zealand. Most articles were descriptive in nature. Some variations in methods emerged.

**Conclusion:**

PAs are operational in 15 nations; their acceptance appears successful and satisfaction with their care largely indistinguishable from physicians. Findings from this analysis highlight one theory that when patient’s needs are met, satisfaction is high regardless of the medical provider. Areas for further research are identified.

## Introduction

We undertook a systematic, scoping review of the literature on patient satisfaction with physician assistants and physician associates (PAs). The topic is germane to the utilization of PAs as they are employed in some fashion on four continents [1]. The rationale for this activity is their growing presence internationally. As more countries adopt and deploy PAs, the question as to what patients want emerges. A fundamental theory is that no health policy or marketing will overcome prevailing attitudes if patients are unwilling to accept care or are not satisfied with a PA’s care [2]. Furthermore, patient satisfaction is an important component of the healthcare experience because patient agreement is considered a desired outcome of care [3].

The education of PAs across the globe is more similar than different, and outcomes of care diverge little from physicians even when the populations are identical [4–6]. The basis for this review was to clarify working definitions, synthesize the evidence, and establish conceptual boundaries around the topic. A second rationale was to summarize and disseminate the research findings, identify gaps in the literature, and offer suggested undertakings for more clarification on the subject.

The research question is: *Are patients satisfied with care provided by PAs?* The objective was to assess the impact on the patient experience with a PA in a medical care encounter.

A PA, for the purpose of this review, is defined as “a healthcare professional trained in medicine and works as part of a medical team in partnership with doctors to provide healthcare to patients” [7]. The PA movement is global, and their presence is noted across 15 countries [1]. However, the literature is diverse and remains to be synthesized.

The concept of “patient satisfaction” is a regularly used indicator of quality in marketing, as a patient retention measure, and a measure of healthcare quality [3]. Because satisfaction of an encounter can affect clinical outcomes, it can mean a great deal for a wide range of health providers. Where it occurs, it is noted both on inpatient hospital services as well as in medical clinics and physician’s offices. Patient satisfaction is also a concept that is evolving. Observations in the twentieth century focused on whether the patient was “pleased to be cured” [8]. As early as 1971, when the development of the PA was still underway, Rousselot et al. called “public acceptance [of the PA] must be studied fully and evaluated fairly.” [9]. A more contemporary view has emerged. This view is that the quality of the healthcare system as well as the health professional is needed when examining patient satisfaction [10]. Satisfied patients are more likely than unsatisfied ones to continue using healthcare services, maintaining their relationships with specific health care providers, and complying with care regimens [11].

If the contemporary PA movement began in the mid-1960s, a view spanning a half-century of patient satisfaction in regard to PA encounters is timely to understand what the shortcomings are and how experience can be improved. The Donabedian model of quality healthcare stresses that the interface of practitioner performance and patient acceptance is where “maximally effective or optimally effective care is sought.” Further investigation explored whether individual or social preferences define the optimum encounter [3].

## Method

Our analysis follows the scoping review methodology as outlined by Arksey and O'Malley and further refined by Peters et al. [12, 13]. This review was registered at the University of York in 2017 (irss505@york.ac.uk).

An information specialist (MM) developed the strategy for MEDLINE, the primary database. After the method was peer reviewed by library colleagues, the strategy was translated into the other pre-selected databases. Database subject headings and keyword searching were utilized for increased sensitivity. Date limits were from 1968 to 2020. No methodological filters were used. Databases searched include MEDLINE (Ovid), Embase (embase.com), CINAHL Plus with Full Text (Ebscohost), PsycINFO (Ebscohost), CENTRAL (Cochrane Library), and Web of Science (Clarivate Analytics). See supplemental files for search strategies. Citation management, including removal of duplicates, was accomplished with EndNote (Clarivate Analytics). The study was considered exempt from our Institutional Review Board scrutiny.

The question “are patients satisfied with physician assistant/associates” ensured that a broad range of literature was included in this scoping review project. Comprehensive inclusion of the scale and scope of available literature was consistent with contemporary scoping review strategies. Exclusion criteria ensured that the PA alone was evaluated by the patient and not included with other health providers such as nurse practitioners (NPs) or midwives where each type of provider was not distinguishable. The methodology is cataloged in Table 1 and identifies inclusion and exclusion criteria.

**Table 1:** Inclusion and exclusion criteria

Criterion	Inclusion	Exclusion
Time period	1968 to 2019	Studies outside these dates
Language	English	Non-English studies were eligible for inclusion (none found)
Type of article	No limits were placed on the peer-reviewed literature type. Must include PAs as focus.	Exclude if PAs were grouped together with another health professional such as an NP and could not be separately analyzed.
Study focus	Patients were asked or surveyed as to how they regarded their care or experience with a PA. The definition of a PA includes those formally trained as a PA.	
Population and sample	Patients that had an encounter with a PA.	Other health professionals if they were merged together with PAs, e.g., NPs, nurses, and technicians.

*Covidence* (Covidence.org), an online systematic reviewing platform, was the software used to screen, review, and select studies. Two reviewers independently screened the title and abstract and reviewed the full text. Each was blind to the other’s decision. A consensus strategy for inclusion was created *a priori* with a third party if one could not be reached. When 11 differences were found, these were resolved without an arbitrator. Data charting was performed with Excel (Microsoft).

**Fig. 1:**
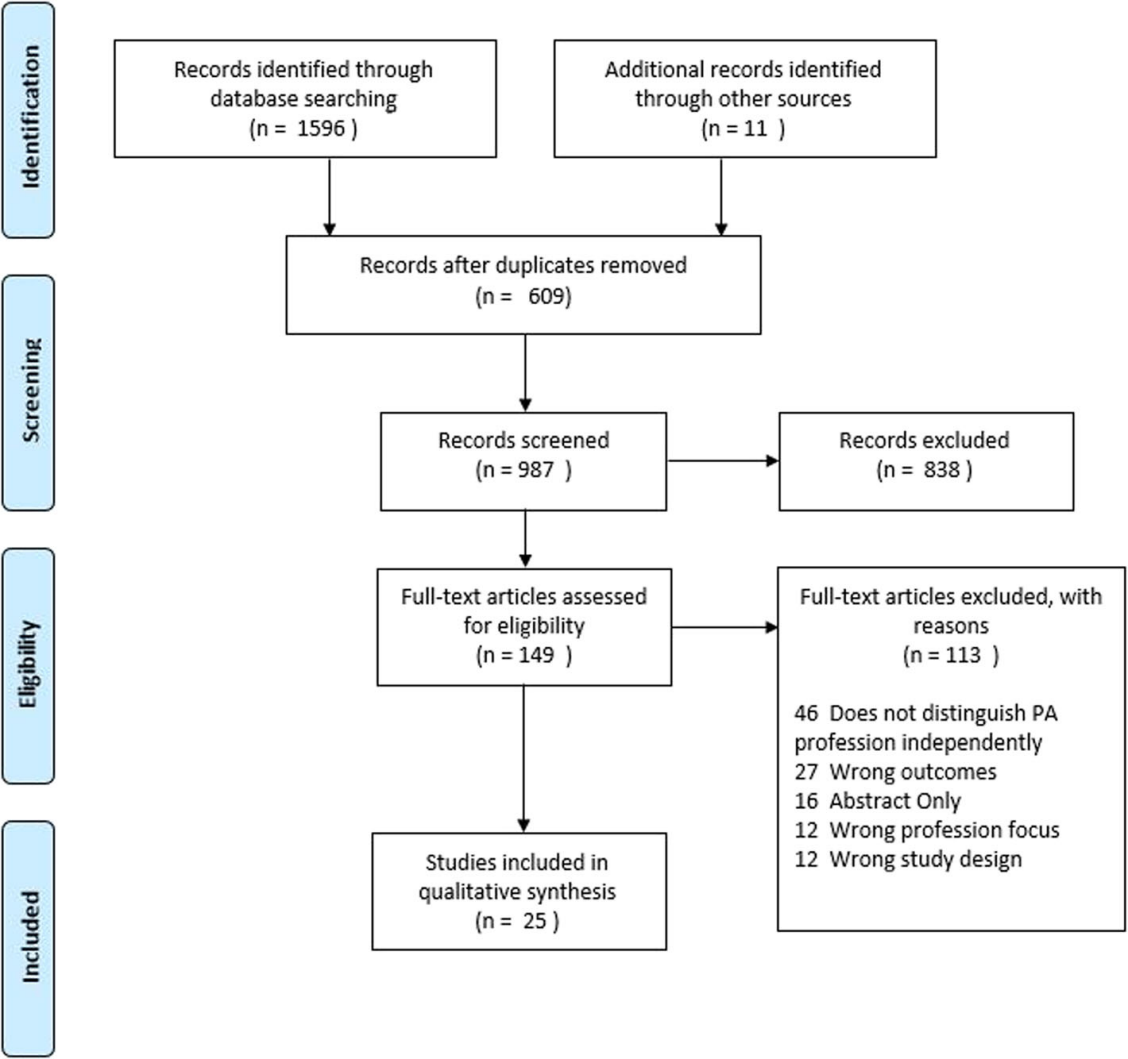
Overview or schematic of the review process

During the article selection, over 95% of articles reviewed were excluded when the criteria in Table 1 were applied. Examples excluded were poster abstracts or papers where specifics on the data-gathering strategies were not detailed. Another small but important percentage of articles reported on patient satisfaction with a PA and NP in the aggregate but did not separate out the two providers. We corresponded with five authors requesting more granular data, but none were forthcoming. After final eligibility filtering, 25 studies were included in this scoping review (Fig. 1).

## Results

In total, there were 987 papers or reports that were identified through database searching. Additional records were identified using “snowball” methodology-reviewing references (*n* = 11). From this effort, 25 articles emerged from the filtering process for final inclusion. These were grouped by national origins for the first analysis (Table 2).

**Table 2:** Geographic regions where patient satisfaction studies on PAs were undertaken

Ireland, Republic of	1
Netherlands, the	1
New Zealand	1
United Kingdom (3 England; 1 Scotland)	4
United States of America	18
Total	25

Articles included in the final review were identified by country of origin to enable a comparative analysis of physician assistant/associates. The majority was American (*n* = 18); four were from the United Kingdom; and one each from Ireland, the Netherlands, and New Zealand. No patient satisfaction studies were reported on PAs in other countries, or on clinical associates of South Africa (Table 2). Only one study was a national survey of patient satisfaction of the elderly—and compared PAs, NPs, and doctors alongside each other [14]. No differences were found between the three types of providers, and at the same time, patient satisfaction with each type of provider was found to be consistently high.

The method of assessment was examined (Table 3). Eight studies reported anonymous surveys administered by mail and six used standardized and validated survey instruments (e.g., Meijer and Drennan) [15, 16]. Older studies, many made at an early stage of PA development, were done by phone or the patient was interviewed in person (e.g., Litman and Farmer) [17, 18]. Anonymous surveys began appearing in the late 1980s.

**Table 3:** Methods of assessing patient satisfaction with PAs

Patient interview in person	5
Patient interview by telephone	4
Chart review	3
Open survey, paper	3
Anonymous survey	8
Pre-visit–post-visit (before–after)	2
Total	25

We analyzed the setting of the patient being seen by a PA. While a quarter were in some type of physician’s office (*N* = 6) or clinic (*N* = 4), six were in specialty clinics such as diabetes or orthopedics (Table 4). Two of the early studies took place in a rural America clinic site. Because many of the early studies investigating PAs were efforts to enhance healthcare delivery as such PAs were viewed more as a complement to physicians than replacements.

**Table 4:** Types of setting where patient satisfaction with PAs was assessed

Hospital in-patient	3
Physician’s office	6
Clinic (military, hospital, community, other)	4
Emergency department (includes urgent care/fast track)	3
Rural clinic	2
Specialty clinic (orthopedic, general surgery, diabetes, hypertension, pediatrics)	6
Mixed settings	1
Total	25

A summary of the included studies is listed in Table 5. All studies were undertaken between 1970 and 2017. Spanning 23 studies, the number of patients interviewed about PAs was at least 8062 (Mean = 350; Max = 1159; Min = 20). However, two studies did not identify the total number of patients surveyed. The total number of PAs assessed spanning all studies was more than 2234, although the accumulative number of PAs is not known because in eight studies the exact number of PAs assessed was not mentioned.

**Table 5:** Summary of scoping review

Author (last name of first author and year)	Location (country, state, and city if known)	Year of data	Study design or methodology	Findings, notes, critique, etc.
Litman 1972 [17]	USA—Iowa and Minnesota	1970	Telephone Survey/interview by research staff following a visit to a PA. *N* = 253 households. Site: rural hospitals, PA specialty unspecified. PAs: N/A (general perception study of what the patient thought about the PA). Q: What is the public perception of the PA?	In total, 2/3 interviewed indicated a willingness to see a PA, 16% would not see a PA. The patient’s view relies heavily on endorsement of the physician and less agreement over specific services; 94% OK with PA taking history and physical examination, 76% in favor of ER procedures. Greatest opposition of PA was in maternity care. One third (34%) were unwilling or very unwilling to be screened by PA if they could see a physician instead. But 83% were okay with the PA referring them to specialist.
Strunk 1973 [19]	USA—California (Los Angeles)	NS	Attitude scale of 30 items. *N* = 300 patients waiting in the clinic Site: outpatient clinic. PAs: N/A (general acceptance study of what the patient thought about the PA) Q: Are PAs acceptable to patients?	Acceptance was higher in patients with some exposure to college, middle class, non-married; 67% overall agreed PAs are a way to improve nations’ health. In fact, 41% responded that PAs should be doing more than routine visits; 67% willing to be seen by a PA “if they felt they knew what they were doing”.
Nelson 1974 [20]	USA—Upper New England	1972	Patient satisfaction questionnaire: *N* = 449 patients Site: 18 outpatient primary care clinics. PAs: 18 Medex trainees Q: How satisfied are patients with the services provided by (Medex) PAs?	A sample of 900 patients seen in 18 practices of upper New England PCPs with a questionnaire. When asked about their opinions of PAs no demonstrable differences were found between respondents and non-respondents; 372 had experience with MEDEX trained PAs, 77 did not. In total 91% were satisfied with the encounter (99% very/somewhat, 87%/100% very satisfied/somewhat) with the physical examination. A total of 89% found the PA very competent; only 1% reported incompetent or not confident in them.
Komaroff 1974 [21]	USA—Massachusetts (Boston)	1970–1972	Protocol-driven diabetes and hypertension management was reviewed by physicians. *N* = 441 patients Site: medical clinic PAs: Number not reported Q: Does the level of care provided by PAs meet patient acceptance?	Patients were randomly assigned to “health assistant” functioning as a PA. In total 441 patient visits were studied for protocol adherence by the HA. Of 286 patients assigned, 6% declined to be routed into the protocol system. Of 53 patients who were told they did not have to see the doctor, only 1 asked to be seen; only 2.2% of total visits did patients seek medical attention in between visits.
Charles 1974 [22]	USA—California (San Francisco)	1972 and 1973	Case study; PAs using clinical algorithms. *N* = 1159 visits spanning one year. Site: VA “drop-in” clinic. PAs were assessed after 3 months of working in the VA. Data collected on site. PAs: 5 Q: Do patients accept care by a PA?	Two separate studies were undertaken; each looked at data recorded by a doctor vs. a PA; patient’s acceptance of the PA was 99% (only 2 patients refused to see the PA). One comment was that the PA spent more time with the patient than physicians.
Maxfield 1975 [23]	USA—New England -Maine, New Hampshire	1974	Questionnaire (paper) sent to patients who had been seen in an emergency room staffed with PAs. *N* = 237 patients Site: Emergency Department PAs: 3 Q: Are patients satisfied with PA care in the ED?	91/237 questionnaires were received from patients (38%). With no exception all reported high satisfaction with PAs.
Storms 1979 [24]	USA—Maryland (Baltimore)	1975	Patient satisfaction questionnaire: *N* = 449 patients Site: 18 outpatient primary care clinics. PAs: 18 Q: Do patients accept care provided by a PA?	Only 4.1% of the sample had seen an NP or PA; overall acceptance was similar between both groups, though slightly favored to NPs.
Jolly 1980 [25]	US (national)	1976, 1977	Quantitative and qualitative survey of Air Force service members and families. *N* = patient number is unclear Site: 4 base military treatment facilities PAs: 23 Q: Are patients satisfied with PA care on a panel system?	Three levels of satisfaction reported. Acceptance and satisfaction were high throughout. The percentage of respondents who felt the PA could handle specific problems declined depending on the complexity of the problem.
Smith 1981 [26]	US—Iowa	1979	Sixteen question survey of patients following visit with PA. *N* = 196 completed Site: Multi-specialty clinic PAs: 4 Q: Are patients satisfied with care provided by a PA?	In total, 92% always or usually satisfied with care by the medical team, 78% usually or always satisfied with team approach. Patients are almost always willing to be seen by the PA if they know the physician is supervising the PA.
Hla 1983 [27]	US—North Carolina	NS	Patients interviewed by a nurse following a visit to the clinic where a PA was seen. *N* = 191 patients. Site: General outpatient clinic, hypertension patients. PAs: 1 Q: Are patients satisfied with care provided by a PA on a medical team?	Compared 191 patient visits with PA management and 200 without PA management of blood pressure. Similar patient characteristics. No significant differences in patient satisfaction.
Oliver 1986 [28]	US—Iowa	1984-1985	Patient interview and questionnaire (hybrid model) following a visit to a PA. *N* = 308 patients. Site: Seven outpatient clinics and two satellite clinics, primary care. PAs: 11 Q: Are patients satisfied with care provided by a PA regarding competency, interpersonal skills?	The questionnaires explored satisfaction, competency, interpersonal skills, time on a 1-5 Likert scale. The results: 4.81 mean for interpersonal skills, 81.5% completely satisfied; PA competency similar at 4.6 mean; 4.48 mean completely or satisfied with time. Females, patients with higher education, and those with more contact with PAs tend to rate PAs higher than all others.
Brady 2004 [29]	US—Oregon (Forest Grove)	2004	Interviews and patient questionnaire, using a validated survey at time of exit from clinic. *N* = 100 patients. Site: Outpatient clinic, family medicine. PAs: 2 Q: Are patients satisfied with care provided by a PA?	Overall, 73% of patients generally satisfied with the care from PA; *N* = 2 PAs; Overall satisfaction was 4.757/5. In total 94/100 of those interviewed gave a 4 or 5 for overall satisfaction of the encounter with the PA.
Hooker 2005 [14]	US—National	2000 = 2001	Cross-sectional survey of Medicare (> 64 years old) patients who received care from a doctor, PA or NP. *N* = 146,880 completed surveys Clinic/specialty unspecified. PAs: 2234 Q: Are patients satisfied with care provided by a PA?	Through a series of survey questions, 95% of all beneficiaries (elderly) said they were happy with their provider regardless of type (doctor, PA, NP). Overall most (95%) said there was little or no problem to find a provider that they were happy with.
Rodi 2006 [30]	US—New Hampshire	2004	Pre- and post-visit surveys with PA or doctor *N* = 87 completed (pre), and 91 completed (post) Site: Outpatient, fast-track clinic PAs: Number not reported Q: Are patients satisfied with care provided by a PA?	In essence this study demonstrates that a fast-track unit staffed by PAs can improve patient satisfaction and decrease LOS. The primary driver is LOS. The patient’s perception of the PA improved when the LOS was shorter.
Farmer 2008 [18]	UK—Scotland	2006–2008	Patient interviewed by a researcher as they exited the clinic where a PA was seen. *N* = 20. Site: Multiple settings and specialties. PAs: 15 Q: Are patients satisfied with care provided by a PA?	All 20 patients interviewed were satisfied with the treatment they had received in the setting that day (very satisfied: 11; satisfied: 9). Four specifically emphasized high satisfaction with the PA. Twelve thought that they had received faster service than usual and 8 thought that the speed of the service was similar to what they would normally receive. Where service was faster, some patients attributed this to the involvement of PAs.
Roy 2008 [31]	US—Massachusetts (Boston)	2005–2006	Retrospective cohort study, Press-Gainey survey determining satisfaction *N* = 992 Site: PA/hospitalist service vs. house staff service (without PA) *N* = 4202. PAs: 3 Q: Are patients satisfied with care provided by a PA as part of hospitalist team?	Patients were similarly satisfied with their care on the PA + Hospitalist service as on the house staff services without the PA. The study did not directly ask patients about PAs. Patients were similarly satisfied with care on PA-hospitalist service when compared with house staff only service.
Dhuper 2009 [32]	US—New York (Brooklyn)	1998–2000	Satisfaction survey questionnaire administered monthly to a of *N* = 1000 patients convenience sample. Site: Inpatient hospitalist service. PAs: 23 Q: Are patients satisfied with care provided by a PA on a medical team?	Comparison of PA/hospitalist vs. Resident/hospitalist models; 95% of patients satisfied with care by providers during 1998-2000 (PA/hospitalist) compared with 96% from 1996-1998 (Resident/hospitalist.). Findings were not statistically significant.
Tataw 2011 [33]	US—California (Los Angeles)	2002–2004	Telephone interviews with parents of children who received general medical and sub-specialty care. The questionnaires explored satisfaction, competency, interpersonal skills, and time on a 1-5 Likert scale. *N* = 71 Site: Pediatrics clinical service, primary care and sub-specialty. PAs: Number not reported Q: Are patients as satisfied with care provided by a PA as a doctor?	Satisfaction measured between PA and doctor. No statistically significant differences in satisfaction scores. Satisfaction, based on summated scores, revealed that parents were slightly more satisfied with services provided by a PA than a doctor.
Berg 2012 [34]	US—Kansas	2007–2008	Prospective, cross-sectional study using telephone surveys of recently discharged level I (emergent) and level II (urgent) trauma patients 4 weeks upon discharge. *N* = 251 Site: ER/Trauma PAs: Number not reported Q: Are patients satisfied with care provided by a PA?	Overall satisfaction 5.04 with PA (Likert scale 1-6). Findings were divided between interpersonal care and technical care. Patients more likely to indicate satisfaction in other areas if satisfied with interpersonal care.
Drennan 2014 [35]	UK—England	2011–2012	Mixed methods—interviews and surveys specific to patient satisfaction. *N* = 539, 34 interviews. Site: Primary care PAs: Number not reported specific to satisfaction Q: Are patients satisfied with care provided by a PA?	Patients and relatives described PAs as positively as the GP. Many of the respondents did not understand who and what a PA was, often mistaking them for doctors.
Appleton-Dyer 2015 [36]	NZ (South Island)	2013–2015	Paper surveys were collected at the time of visit from sites where a PA was part of a demonstration project. *N* = 511. Site: Outpatient clinics, primary care and urgent care “drop-in” clinics. PAs: 26 Q: Do patients accept PAs and are patients satisfied with care provided by a PA?	In total 220 surveys identified the PA. There were no significant differences at the 0.05 level between the PA and any other health professional. This suggests that patients are just as satisfied with the care they receive from a PA as they are with other health professionals.
Johnson 2016 [37]	US	NS	A patient satisfaction survey was distributed to patients receiving care from PAs at the time of the visit. *N* = 87 Site: Outpatient orthopedic clinic. PAs: Number not reported Q: Are patients satisfied with care provided by a PA?	Waiting time, technical skill, interpersonal manner and overall satisfaction with the PA was 6.9 (on 7-point Likert scale)
Meijer 2017 [15]	NL—Friesland	2015	European Union standardized Consumer Quality Index - mailed form sent to the patient within 2 weeks of an encounter at a physician’s office or clinic. *N* = 92 for physicians, *N* = 110 for PAs Site: Outpatient clinics, primary care. PAs: Number not reported. Q: Are patients satisfied with care provided by a PA vs a doctor?	For the most part patients were as satisfied with PAs as they were with physicians. The one exception is that female patients seen by GPs were less satisfied than with PAs. The gender of the PA or physician was not collected. PA and physician visits were compared.
Drennan 2019 [38]	UK—3 regions	2016–2017	Patient interviews about their experience with a PA. *N* = 28 Site: Six hospitals, acute care PAs: 43 Q: What is the impact of PAs on the patient experience?	“Patients and relatives described PAs positively, but most did not understand who and what a PA was, often mistaking them for doctors”
Joyce 2019 [39]	IR—Dublin	2017	Patient satisfaction survey. *N* = 74 completed surveys; 22 seen by PAs and 52 by doctor Site: Outpatient hospital clinic PAs: 4 Q: Are patients satisfied with care provided by a PA?	Satisfaction with care survey -- no difference in the patient satisfaction ratings between PAs and doctors

Note: *IR* Ireland, Republic, *PA* physician assistant/associate, *PCP* primary care provider, *VA* Veterans Administration [or Veteran Affairs], *NL* the Netherlands, *NZ* New Zealand, *NS* not stated in the manuscript, *LOS* length of stay

The majority of studies (*N* = 23) was descriptive in reporting. In two articles, a mixed-methods approach was incorporated. In most studies, the research question probed the patient’s satisfaction with the PA. In the remainder, it was explored as a secondary outcome. Older articles focused more on patient acceptance instead of satisfaction, likely due to the emerging concept of the PA as a new profession. Early studies describing patient acceptance were included in this review because they were interpreted by the authors as satisfaction and willingness to be seen (e.g., Strunk 1973).

Overall, patient satisfaction compares favorably with physicians in this scoping review. This finding was consistent through all 25 studies and, where a comparison was made, patient satisfaction ranged from 94 to 100% regardless of the instrument used. Exit interviews (patients interviewed in person) did not differ significantly from anonymous paper interviews. No assessment via electronic communication was done in the 25 studies we assessed.

## Discussion

The majority of studies on patient satisfaction came from the United States (18/25), reflecting over 50 years of experience with PAs. It should be noted that analysis across five countries finds remarkably consistent results—patients were generally satisfied with PAs regardless how the encounter or experience was assessed.

Early development of US PAs (including the MEDEX model and the Child Health Associate model) was aided by federal and foundation grants intended to examine if these new health professionals were going to meet the needs of society. Patient awareness and acceptance with PAs was a cornerstone of health policy research. In the US, the acceptance with PAs by patients in theory or in practice consistently grew as reflected in patient satisfaction studies. This concept mirrors our findings in the articles we analyzed, as early studies focused on acceptance, which evolved into a focus on satisfaction in more recent studies. Similar findings, using more refined survey instruments, found that the contemporary PA in the UK, IR, NL, or NZ, was widely accepted. More specifically, patients were as satisfied with PAs as the doctor [15, 35]. None of the studies included in this review found patient dissatisfaction beyond single digit percentage.

Techniques in how patients were assessed for their satisfaction varied widely. This included exit interviews (as the patient left the clinic), chart reviews, telephone surveys, anonymous paper surveys, and household surveys in rural areas. Only a few of the surveys were standardized and validated (e.g., Medicare survey of the elderly, European Union Consumer Quality Index), and only one involved a pre-visit survey followed by a post-visit survey. A few of the studies matched the PA and the patient for age, gender, race, ethnicity, or other characteristics. The US Medicare study was unique as it was national in scope. Some studies looked exclusively at patient satisfaction with care provided by a PA, though some made direct comparisons with other health professionals. Of the 25 studies analyzed, none showed that patient satisfaction with the PA was significantly less than the doctor. In the one study where PAs and NPs were compared, the satisfaction results were found comparable. There are a number of studies on patient satisfaction that include PAs folded in with NPs. Such studies are important in their own right as they inform consumers and policy makers but were excluded from this study because they failed to discriminate between the two providers where some differences might exist.

In addition to differing assessment techniques, there were other reasons why direct comparisons between studies were difficult. These factors included variability in study types, size of studies, patient populations, settings, specialties, assessing individual providers vs. teams, utilization/role with PAs, and scope of practice. Furthermore, some studies asked about satisfaction directly whereas others made inferences. Due to the wide date ranges between studies, PAs in earlier studies were less recognized as a profession than in later reports. Early demonstration projects viewed PAs more as doctors’ assistants while contemporary PAs are seen as medical professionals similar to doctors. This is reflected in the studies done by Litman [18], Nelson [20], and Hla [27]. The European studies assumed the PA was filling an otherwise deemed physician role [15, 35]. Spanning the 50+ years of PA utilization, the adherence to protocols to treat patients has been replaced by “best practice” or community standards of care.

Notably, in this study, it is difficult to directly compare several studies as the scope of the PA in early studies was different than in later studies. In some earlier studies, patients were either satisfied or accepted care by a PA but in a very limited scope of practice. Implications were made that they may not have accepted care if a PA had increased responsibility in which they did not feel a PA had adequate training. This is also due, in part, to the lack of knowledge about the PA profession at the time the studies were conducted. In later studies, as the profession became more well known, satisfaction encompassed the current scope of PAs.

Measuring patients’ perception of care is important for a number of reasons. The first is that no amount of observation science could overcome a negative perception if patients refused to accept the role of the PA. In addition, satisfaction correlates with compliance, health outcomes, and patients returning to see the same provider. Patient satisfaction surveys are essentially assessing service delivery based on the patients’ viewpoints of the organization as well as the provider of that service. Outcomes are improved when the patient and the medical clinician correlate with patient satisfaction, quality of life, compliance with instructions, and most importantly, health outcomes [40, 41]. Patient satisfaction also correlates with continuity of care and likelihood of returning to the provider for longitudinal care [42, 43].

### Recommendations

While this review found a significant body of literature on the subject of patient satisfaction with PAs, 72% was produced in the United States. As more countries expand their observations of PA behavior, the number of patient satisfaction studies is expected to grow. We believe that all countries should have some fundamental understanding how well and to what extent their citizens need and accept their providers of medical care.

In undertaking this project, a wide assortment of studies was examined (although not necessarily included). From this body of patient satisfaction literature, we identified variables we believe need to be introduced into the research model when testing the hypothesis that patients are largely accepting and, in general, satisfied with PAs in terms of care, experience, and outcomes. These are:

- Gender—A match for gender between provider and patient should assess and compare where there is a difference. In the case of children, the gender of the parent or guardian should be known as well as the provider.
- Race and ethnicity—A match for race and ethnicity needs to assess if the differences found in patients and physicians change with patients and PAs.
- Age—The four broad age groups needed for examination are children, 18–40, 40–65, and > 65. Younger versus older providers are broad areas that need to be compared in provider-patient encounters.
- Medical specialty—PAs in hospitals, clinics (urban and rural), physicians’ offices, emergency rooms, urgent care clinics, and orthopedic clinics were assessed. Because the majority of PAs in the US, UK, NL, and Canada are not in primary care settings, the roles, specialties, and settings not assessed compile a large list.
- Country—All countries should undertake patient satisfaction studies and results compared across borders to see where areas of improvement can be made. This is not only for PAs but also for the wide range of health professionals.

### Limitations

The greatest limitation to this study is the criteria we applied: that the included study needed to be peer reviewed and published. By this gauge, a large number of studies on patient satisfaction were not included. This is due to the observation that many health organizations undertake patient satisfaction studies for enrollment and marketing concerns but do not publish their results or even make the results publicly known. We were aware of these studies, some by marketing companies for commercial purposes, but including them would be outside the scrutiny of scientific study and scoping review criteria.

## Conclusion

The contemporary physician assistant/associate emerged in the 1960s and now occupies a role across a wide set of societies. We asked the question: *Are patients satisfied with care provided by a PA?* Using the scoping review format, almost 1000 mentions of some aspect of PAs and patient satisfaction were screened; 25 met criteria for inclusion. Of those analyzed, 18 were US studies and three were from the UK. The settings ranged widely from small rural clinics to large urban hospitals. In almost all studies comparing PA care to physicians, the patients made little if any distinction between the two. In this scoping review, it appears that patients are satisfied with PA-led care. The next phase of patient satisfaction research should compare provider and patient race, age, and include a diverse type of setting and medical specialty.

## Data Availability

All data and material are available for inspection and confirmation. All references are published literature.

## Abbreviations

CAs: Clinical associates of South Africa
IR: Ireland, Republic of
MD: Medical doctor/physician
NL: The Netherlands
NP: Nurse practitioner
NS: Not stated
NZ: New Zealand
PAs: Physician assistants and/or physician associates.
PCP: Primary care provider
VA: Veterans Administration [or Veteran Affairs]
